# Antitumor effect of an adeno-associated virus expressing apolipoprotein A-1 fused to interferon alpha in an interferon alpha-resistant murine tumor model

**DOI:** 10.18632/oncotarget.14127

**Published:** 2016-12-23

**Authors:** Marcos Vasquez, Vladimir Paredes-Cervantes, Fernando Aranda, Nuria Ardaiz, Celia Gomar, Pedro Berraondo

**Affiliations:** ^1^ Program of Immunology and Immunotherapy, Center for Applied Medical Research (CIMA), Navarra Institute for Health Research (IdiSNA), Pamplona, Navarra, Spain; ^2^ Centro Médico Nacional La Raza, IMSS, México DF, Mexico

**Keywords:** colorectal cancer, liver metastasis, cancer immunotherapy, T regulatory cells, PD-1

## Abstract

Interferon alpha (IFNα) is a cytokine approved for the treatment of several types of cancer. However, the modest effect on overall survival and the high toxicity associated with the treatment has reduced the clinical use of this cytokine. In this study, we have developed a tumor model that reproduces this clinical setting. A high dose of an adeno-associated virus encoding IFNα (AAV-IFNα) was able to eradicate a liver metastases model of colon cancer but induced lethal pancytopenia. On the other hand, a safe dose of AAV-IFNα was not able to eliminate the liver metastases of colon cancer. In this IFNα-resistant tumor model, administration of an adeno-associated vector encoding apolipoprotein A-1 fused to IFNα was able to fully eradicate the tumor in 43% of mice without toxicity. This antitumor effect was limited by suboptimal long-term CD8^+^ T cell activation and the expansion of T regulatory cells. In contrast, IFNα upregulated suppressor molecules such as PD-1 and interleukin 10 on CD8^+^ T lymphocytes. In conclusion, we show that apolipoprotein A-1 fused to IFNα is a novel antitumor drug that differs from IFNα in the modulation of suppressor mechanisms of the immune response. These differential properties pave the way for rational combinations with other immunomodulatory drugs.

## INTRODUCTION

Several pattern-recognition receptors expressed on tumor and stromal cells trigger the release of interferon alpha (IFNα) upon binding of danger-associated molecular patterns. Initially, the production of IFNα was attributed to several toll-like receptors such as TLR-3, TLR-9, TLR-7 and TLR-8 [1]. Recently, research has highlighted the role of cytoplasmatic RNA (RIG-1 and MDA-5) and DNA receptors (the STING pathway) in the release of IFNα in tumor cells [2, 3]. IFNα can be produced by endogenous stimuli [4] but can also be induced by several conventional tumor treatments such as chemotherapy and immunological therapy [1, 2, 5]. The potent antineoplastic activities observed *in vitro* and in a variety of animal models led to the initiation of clinical trials. As a result, IFNα monotherapy was approved for a number of indications [6]. However, the low efficacy and systemic side effects have limited its clinical utility. During high-dose or long-term IFNα therapy, patients suffer high-grade side effects that include fatigue, fever, headache, muscle aches, nausea, dizziness, anorexia, depression, and leucopenia. In a significant number of cases, such side effects lead to the discontinuation of the treatment [7]. An alternative dosing schedule of continuous, low-level delivery, rather than intermittent, high concentration pulsed-dosing, could be achieved by gene therapy. Ideally, IFNα gene therapy might avoid the toxicity of interferon while maintaining its antitumor efficacy. The adeno-associated virus (AAV) is a natural replication-defective single-stranded DNA parvovirus. The lack of pathogenicity of the virus, its persistence, long-term expression and relative lack of immune response make this vector an appropriate tool for our goal. Several clinical trials have demonstrated the safety, efficiency, and efficacy of these vectors [8]. To further improve the pharmacokinetic properties of IFNα, we have developed a strategy based on its fusion to apolipoprotein A-1 (ApoA1). ApoA1 is produced in the liver and incorporated into high density lipoprotein. Then, the high-density lipoproteins circulate interacting with all the cells of the organism through SR-B1, picking up the cholesterol from them. Finally, the high-density lipoproteins are internalized and catabolized in the liver. Therefore, ApoA-1 fusion proteins take advantage of the interesting pharmacokinetic properties of high-density lipoproteins. The fusion protein of IFNα and ApoA-1 has a longer half-life in the circulation and the hematological toxicity is reduced, likely through a reduction of the cytotoxic effect of the IFNα fused to ApoA-1 [9].

Metastatic liver cancer is a life-threatening condition frequently observed in colorectal cancer patients. Hepatic lesions are found in 10% to 25% of cases at the time of diagnosis, and 30% of patients have no evidence of dissemination to any other organ. In addition, recurrence after surgical resection of the colorectal tumor occurs mainly in the liver, with a 20-25% rate of metachronous liver metastases. Current therapeutic strategies are far from satisfactory and it is clear that new therapeutic options are needed to improve the clinical management of hepatic metastases from colon cancer [10].

In this study, we tested the antitumor activity of long-term expression of IFNα alone or fused to ApoA1 using an AAV vector in a murine model of hepatic colorectal metastasis after liver implantation.

## RESULTS

### Therapeutic window of AAV-IFNα

**Figure 1:**
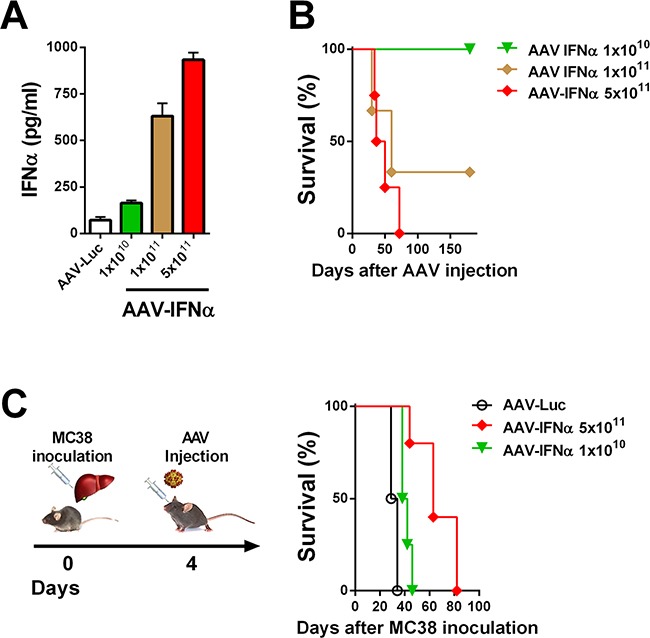
Lethal doses of IFNα are able to eradicate a liver metastases model of colon cancer **A**. IFNα serum levels in mice were determined by ELISA one week after being treated by different doses of AAV-IFNα (*n* = 3, mice per group). **B**. Kaplan-Meier plot representing the survival of mice treated by different doses of AAV-IFNα (*n* = 3, mice per group). **C**. Schematic representation of a mouse model for liver metastases from colon cancer (left panel). Colon cancer cells (MC38) were injected into the liver of mice and, four days later, different doses of AAV-IFNα were intravenously administrated (*n* = 6, mice per group). Survival of mice is represented in a Kaplan-Meier plot (right panel).

In order to find a safe dose of an AAV encoding IFNα, C57BL/6 male mice were intravenously injected with different doses of an AAV8 expressing IFNα under the transcriptional control of the constitutive and ubiquitous elongation factor-1α promoter (EF1α) AAV-IFNα, 5 × 10^11^, 1 × 10^11^ and 1 × 10^10^ viral genomes (vg). A control group was injected with 5 × 10^11^ vg of an AAV8 expressing luciferase under the control of the same promoter. Seven days after vector administration, IFNα expression in serum was analyzed by ELISA. The IFNα levels in plasma were vector dose-dependent (Figure 1A). Next, we analyzed the survival of the mice treated with the different vector doses. All mice receiving the high dose of AAV-IFNα had to be sacrificed by day 60 due to clear signs of ill health as evidenced by significant weight loss. The medium dose was also lethal in 77% mice while all the mice treated with the low dose survived without any signs of toxicity (Figure 1B).

### Lethal doses of AAV-IFNα are required to eradicate liver metastases of MC38 colon carcinoma

**Figure 2:**
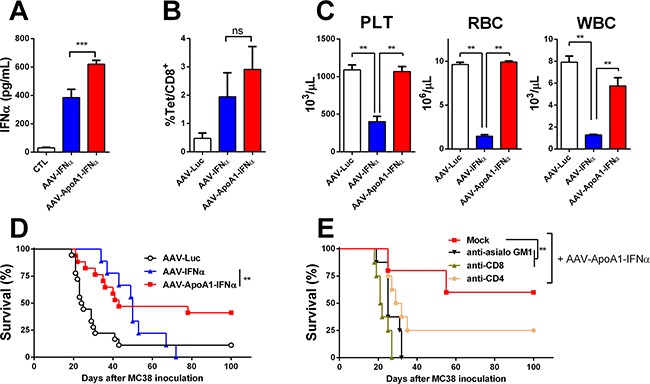
AAV-ApoA1-IFNα is able to eradicate tumors without lethal toxicity IFNα levels **A**. and MC38 tetramer-specific CD8^+^ T cells **B**. were determined in peripheral blood one week after intrahepatic injection of 5×10^5^ MC38 cells followed by intravenous administration of AAV-Luc, AAV-IFNα or AAV-ApoA1-IFNα (5×10^11^ vg) four days later (*n* = 5, mice per group). **C**. Peripheral blood platelets (PLT), leukocytes (WBC) and red blood cell (RBC) counts were analysed at 30 days after AAV-Luc, AAV-IFNα or AAV-ApoA1-IFNα administration to tumor-free mice (*n* = 3, mice per group). Kaplan-Meier plot representing the survival in a liver metastases model from colon cancer treated at four weeks with AAV-Luc, AAV-IFNα and AAV-ApoA1-IFNα (AAV-Luc *n* = 18; AAV-IFNα, *n* = 9; AAV-ApoA1-IFNα, *n* = 17) **D**. or treated with AAV-ApoA1-IFNα and NK or T cell depleting antibodies at day 4, 6, 11 and 13 after MC38 injection (Mock *n* = 6; anti-CD4, anti-CD8 and anti-asialo GM1 *n* = 8) **E**. Data were analysed by one way ANOVA, followed by the Bonferroni multiple comparison test (A, B and C) **P < 0.01 and weighted log-rank test with the Fleming–Harrington class of weights (D) **P < 0.01.

We established a liver metastasis model of colon cancer by direct implantation of 5×10^5^ MC38 cells. Four days after cell implantation, mice were treated intravenously with two different doses of AAV-IFNα: 1×10^10^ vg and 5×10^11^ vg or AAV-Luciferase. At day 21 after virus injection, tumor volume was analyzed by abdominal echography. All the animals from the control group and from the low dose AAV-IFNα group developed tumors while none of the animals receiving the highest dose of AAV-IFNα developed tumors (data not shown). Survival was checked daily and mice were euthanized if their general status deteriorated. Fifty days after cell implantation, all the animals from the control group and the low dose AAV-IFNα group died as a consequence of tumor progression. The animals receiving AAV-IFNα at a high dose died without any tumors in the liver, presumably due to profound pancytopenia (Figure 1C and Figure 2C).

### Safety and antitumor effect of an AAV encoding apolipoprotein A-1 fused IFNα

Next, we analyzed whether the expression of apolipoprotein A-1 fused to IFNα could attain an antitumor effect in this IFNα-resistant tumor model. An AAV8 expressing apolipoprotein A-1 fused to IFNα (AAV-ApoA1-IFNα) under the transcriptional control of the constitutive and ubiquitous elongation factor-1α promoter was constructed. The high dose of 5×10^11^ vg also induced high levels of the cytokine in serum. The levels of IFNα in serum were higher seven days after administration of AAV-ApoA1-IFNα than AAV-IFNα with the same dose (Figure 2A). Furthermore, we compared the induction of MC38-specific CD8^+^ T cells. We inoculated the MC38 cells in the liver, and four days later, mice were treated with the AAV-Luc, AAV-IFNα or AAV-ApoA1-IFNα. One week after AAV injection, MC38-specific CD8^+^ T cells were analyzed in peripheral blood lymphocytes. This tumor-specific population was expanded in mice treated with a vector encoding IFNα or ApoA1-IFNα, indicating that both vectors could enhance T cell-mediated antitumor effector immune responses (Figure 2B).

Consequently, we analyzed the blood cell counts at day 30 after tumor challenge. The group treated with the AAV-IFNα developed profound pancytopenia. In sharp contrast, blood cell counts in mice treated with the same dose of AAV-ApoA1-IFNα remained at baseline levels (Figure 2C).

Next, we compared the antitumor effect in the MC38 tumor model. A significant delay in mice death was observed in those animals treated with AAV-ApoA1-IFNα. Fifty seven percent of mice developed tumor in the liver and had be sacrificed. Remarkably, 43% of mice fully eradicated the tumor and remained alive without any sign of physical deterioration. In mice treated with AAV-IFNα, survival increased but finally, all mice succumbed due to the toxic effect of the high doses of IFNα required to eradicate the tumors (Figure 2D). In order to analyze the main immune effector cells implicated in the antitumor activity of AAV-ApoA1-IFNα, we depleted the CD4^+^, CD8^+^ T lymphocytes or NK cells with appropriate antibodies. Depletion of CD8^+^ T lymphocytes or NK cells completely abrogated the antitumor efficacy while depletion of CD4^+^ T lymphocytes had a partial effect (Figure 2D).

### Immune-related differences between AAV-IFN and AAV-ApoA1-IFNα

**Figure 3:**
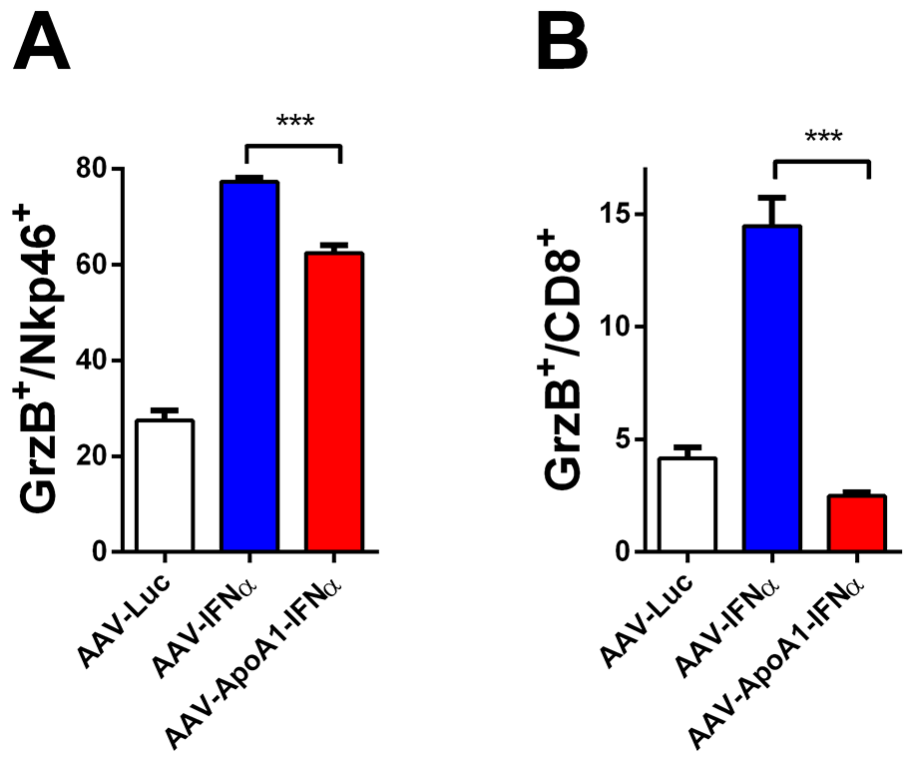
Defect in long-term CD8+ T cell activation after AAV-ApoA1-IFNα in mice The percentages of granzyme B in NK cells **A**. or CD8^+^ T cells **B**. were determined by flow cytometry analysis in spleens of mice two weeks after intravenous administration of AAV-Luc, AAV-IFNα or AAV-ApoA1-IFNα (*n* = 4, mice per group). Data were analysed by one way ANOVA, followed by the Bonferroni multiple comparison test ***P < 0.001.

Having established that AAV-ApoA1-IFNα is able to achieve an immune-mediated antitumor effect in a difficult to treat animal model, we then sought to better understand the differences between both viruses. To this end, we analyzed the immune response induced in non-tumor bearing mice two weeks after virus administration, when the tumor-specific T lymphocytes normally decline. We found that while granzyme B (GrzB) was activated to a similar extent in NK cells by both viruses (Figure 3A), CD8^+^ T lymphocytes treated with AAV-ApoA1-IFNα barely upregulated this effector molecule which is critically involved in the effector function of cytotoxic T lymphocytes [11] (Figure 3B). Thus, our findings suggest that a partial defect in the long-term activation of the CD8^+^ T cell compartment limited the antitumor efficacy of AAV-ApoA1-IFNα.

**Figure 4:**
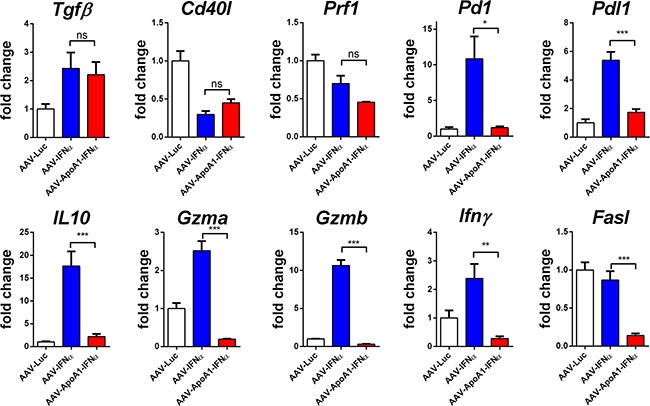
Differences in gene expression induced by AAV-IFNα and AAV-ApoA1-IFNα in CD8+ T cells Gene expression was determined by quantitative real time PCR in CD8^+^ T cells isolated from spleens of mice two weeks after intravenous administration of AAV-Luc, AAV-IFNα or AAV-ApoA1-IFNα (*n* = 4, mice per group). Data were analysed by one way ANOVA, followed by the Bonferroni multiple comparison test ***P < 0.001, **P< 0.01, *P< 0.05.

To further analyze this effect, we purified CD8^+^ T cells from mice treated with AAV-Luc, AAV-IFNα or AAV-ApoA1-IFNα for 2 weeks and performed a gene expression analysis of immune-related genes by real-time PCR. Among the genes analyzed, some were modulated by the two vectors expressing IFNα. This was the case for TGFβ, that was upregulated, and for CD40L and perforin 1, that were downregulated. However, differential effects were observed in IFNγ, granzyme A and B and Fas ligand expression, crucial effector molecules for tumor immunosurveillance [12]. The expression of these genes were upregulated by AAV-IFNα but not by AAV-ApoA1-IFNα. As expected for activated CD8^+^ T lymphocytes, several immune-regulatory genes such as PD-1, PD-L1 and IL10 were upregulated in the AAV-IFNα but these genes were not upregulated by the fusion protein (Figure 4). A similar pattern was observed in CD8^+^ T lymphocytes after 1 week of treatment (data not shown).

### Expansion of T regulatory cells by AAV-ApoA1-IFNα

**Figure 5:**
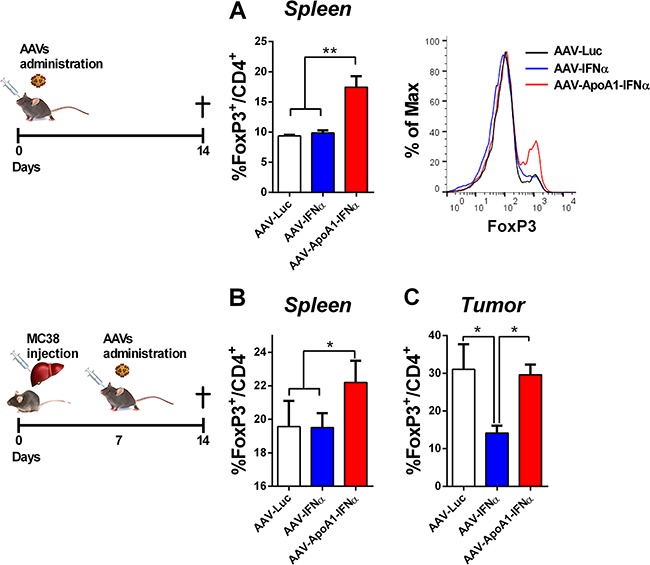
AAV-ApoA1-IFNα increases the FoxP3+ percentage in CD4+ T cells Flow cytometry analysis for the intracellular expression of FoxP3 in CD4^+^ T cells was performed in spleens of mice treated with AAV-Luc, AAV-IFNα and AAV-ApoA1-IFNα for two weeks **A**. Mice received an intrahepatic injection of MC38 and 5 days later were treated with the viruses. One week later, to analyse the FoxP3 in CD4^+^ T cells, flow cytometry was performed in spleen and intrahepatic tumors **B**. (*n* = 3, mice per group). Data were analysed by one way ANOVA, followed by the Bonferroni multiple comparison test **P< 0.01.

From the above experiment, we excluded the possibility that AAV-ApoA1-IFNα induced CD8^+^ T cell intrinsic regulatory mechanisms. Thus, we focused our attention on another important immunosuppressive population that may dampen the long-term activation of T lymphocytes. T regulatory cells are naturally produced to maintain peripheral tolerance and can be induced by stressful conditions such as viral infections and tumors. These CD4^+^ T cells are characterized by the expression of the transcription factor Foxp3 [13]. Thus, we analyzed the percentage of FoxP3^+^ cells in the CD4^+^ T cells from the spleens of the different treatment groups in non-tumor bearing mice. Interestingly, these cells were expanded by the AAV-ApoA1-IFNα but not by the AAV-IFNα, providing a specific regulatory mechanism of the antitumor effect exerted by the AAV-ApoA1-IFNα (Figure 5A).

Finally, we confirmed these results in tumor-bearing mice. The treatment with AAV-ApoA1-IFNα increased the percentage of FoxP3^+^ cells in the CD4^+^ T lymphocytes in the spleen (Figure 5B). In the tumor, AAV-IFNα reduced the percentage of T regulatory cells with a concomitant enhancement of the effect or CD4^+^ T lymphocytes. In contrast, AAV-ApoA1-IFNα retained the percentage of T regulatory cells observed in the tumor (Figure 5C).

## DISCUSSION

IFNα is a potent antitumor agent with direct effects on tumor cells inducing cell cycle arrest, apoptosis or senescence [7]. High doses of IFNα can also alter the angiogenesis required to sustain tumor growth [14]. However, the high doses required to achieve these direct antitumor effects are associated with high-grade side effects [7]. The indirect antitumor effects of IFNα can also be mediated by the activation of an antitumor effector immune response [6]. IFNα is a key cytokine that acts as a signal-3 cytokine in the immunological synapse formed by professional presenting cells, dendritic cells, and T lymphocytes. This signal-3 cytokine modulates signal-1 transmitted by the interaction of the class I MHC molecules and the TCR and signal-2 provided by the co-stimulus receptors [15]. In order to potentiate the immune mediated effect of IFNα, several strategies are currently being tested in clinical trials to achieve a physiologic release of IFNα using molecules that activate toll-like receptors or cytoplasmatic receptors of RNA or DNA [7, 16]. In the present study, we used a gene transfer strategy to achieve local expression of the cytokine. As a transfer vector, we used an adeno-associated viral vector that due to its low immunogenicity allows long-term transgene expression. AAV-IFNα showed levels of the cytokine in serum that were dose-dependent. Mice treated with AAV-IFNα showed a significant expansion of tumor-specific CD8^+^ T cells and the acquisition of effector functions by NK cells and CD8^+^ T lymphocytes. These activation mechanisms were counter regulated by the induction of suppressive molecules in CD8^+^ cells such as PD-1 and IL10. Indeed, blockade of these molecules has been used to potentiate the antitumor effects of IFNα [17, 18]. However, the viral dose required to eradicate tumors led to profound pancytopenia. Tumor growth inhibition after injection of short short-term expression vectors carrying IFNα has been obtained in different animal tumor models [19–21] but we have established an aggressive tumor model that could not be subverted by IFNα reflecting the clinical response in most of the cancer patients treated with IFNα.

We have previously reported that the fusion of interferon alpha and apolipoprotein A-1 limited the cytotoxic effects of IFNα and this effect translates into reduced hematological toxicity *in vivo* [9]. We, therefore, hypothesized that the AAV encoding the fusion protein could eradicate liver tumors without the lethal adverse effects. Indeed, a remarkable antitumor efficacy was achieved with the eradication of the tumor in a high percentage of the tumor-bearing mice in this IFNα-resistant model. We detected an expansion of tumor-specific T lymphocytes 7 days after virus administration and a strong induction of granzyme B on NK cells 14 days after virus administration. It is likely that these immune effector cells contribute to the antitumor effect in cooperation with other mechanisms of action such as the blockade of angiogenesis [14]. In spite of the potential interest of the fusion protein as monotherapy, the lack of tumor eradication in 57% of the tumor-bearing animals points to the need for combination with other immunotherapies. Thus, we explored the differences in the activation of the immune response between IFNα and ApoA1-IFNα. NK cell activation was similar with both compounds and thus, ApoA1-IFNα may be combined with therapies that rely on NK cell activity such as antibodies that induce antibody-dependent cell cytotoxicity. Regarding the CD8-mediated immune response, we have previously reported an enhancement of the cytotoxic activity of T lymphocytes after short-term expression of the fusion protein [9], but we detected a dampened activation of CD8^+^ T lymphocytes with the ApoA1-IFNα two weeks after virus administration. In line with these results, PD-1 was not activated thus precluding the combination with antibodies that block this pathway. The likely explanation for these observations is that the expansion of T regulatory cells observed at day 14 may limit the long-term activation of effector CD8^+^ T cells. Thus, the fusion of apolipoprotein A-1 and IFNα is a safe IFNα derivative with antitumor activities but it must be combined with strategies to keep T regulatory cells in check. In this regard, co-expression of FoxP3 inhibitory peptides [22] or low dose cyclophosphamide [23, 24] could be interesting therapeutic strategies that will be explored in future experiments.

## MATERIALS AND METHODS

### Animal handling

Experiments were performed with 6-8 week-old male C57BL/6 purchased from Harlan Laboratories (Barcelona, Spain). Mice were maintained under pathogen-free conditions and the experimental design was approved by the Ethics Committee for Animal Testing of the University of Navarra.

Recombinant AAV8 vectors were inoculated via retro-orbital injection. Previously, mice were anesthetized by intraperitoneal injection of a mixture of xylazine (Rompun 2%, Bayer) and ketamine (Imalgene 500, Merial) 1:9 v/v.

The murine model of hepatic metastasis from colon cancer involved direct implantation of 5×10^5^ MC38 cells into the left lobe of the liver under isoflurane anesthesia. Survival was checked daily and mice were euthanized if their general status deteriorated.

### Depletion of lymphocyte populations

Mice received four intraperitoneal injection of 15μl of anti-asialo GM1 antiserum (Wako Pure Chemical Industries, Osaka, Japan; Cat. No. 986-10001), 300μg of anti-CD8 (clone 53.6.72) and 200μg of anti-CD4 (clone GK1.5) at day 4, 6, 11 and 13 after intrahepatic injection of MC38. Anti-CD4 and anti-CD8 were provided by Dr. I. Melero (Center for Applied Medical Research, Navarra, Spain).

### AAV vectors

Experiments were performed with AAV serotype 8 expressing mouse IFNα1 or the fusion of ApoA1 and IFNα1 under the transcriptional control of the elongation factor 1α promoter (EF). The AAV were produced by co-transfection of pDP8.ape (PlasmidFactory GmbH & Co. KG, Bielefeld Germany) and pAAV IFNα, pAAV-ApoA1-IFNα or pAAV-Luc plasmids into HEK-293T cells. For each production, a mixture of plasmids, 20 μg of pAAV plasmid and 55 μg pDP8.ape, was transfected into 293 T cells using linear PEI 25 kDa (Polysciences, Warrington, PA, USA). Two days later, AAV was purified from the cell lysates by ultracentrifugation in Optiprep Density Gradient Medium (Sigma-Aldrich, St Louis MO). To titer the AAV productions, viral DNA was isolated using “The High Pure Viral Nucleic Acid” kit (Roche Applied Science. Mannheim, Germany). The concentration of viral particles was subsequently determined by real-time quantitative PCR using primers specific to the EF promoter: Fw: *5′-ggtgagtcacccacacaaagg-3′* and Rv: *5′-cgtggagtcacatgaagcga-3′*.

### Determination of murine IFNα

Serum IFNα levels were measured using a VeriKine™ Mouse Interferon Alpha ELISA Kit (PBL, NJ, USA) following the manufacturer's recommendations.

### Hemogram

Thirty days after the AAV injection, blood samples were collected in tubes with 0.5% heparin (Mayne Pharma, Mulgrave, Australia) as the final concentration. Hemograms were analysed using the Drew Scientific HemaVet Hematology Analyzer (CDC Technologies, Oxford, CT) following the manufacturer's recommendations.

### Cell isolation

Cell suspensions of the spleen were obtained by mechanically disrupting the tissue with a syringe plunger in cold RPMI 1640. Red blood cells were removed using ACK buffer. Splenocytes were washed in cell culture medium (RPMI 1640) and filtered through a 70 μm nylon cell strainer. Cell concentrations were determined with an automatic animal cell counter and splenocytes were adjusted to a desired final concentration.

CD8^+^ T cells were enriched from pooled spleen by anti-CD8 (Ly-2) mAbs (Miltenyi Biotec, Auburn, CA) and separated using the AutoMACS magnetic separation system (Miltenyi Biotec, Auburn, CA).

### Flow cytometry

Cells were incubated for 10 minutes with Fc Block and stained with an optimal dilution of each antibody for 15 minutes at 4°C. For NK cells analyses, cells were stained with anti-NKp46-PE antibody. CD8^+^ T cells were stained with anti-CD8-FITC antibody. Then, cells were fixed, permeabilized, and stained with specific intracellular anti-GrzB-APC antibody. To identify MC38 tetramer-specific CD8^+^ T cells, cells were stained with the iTAg MHC class I tetramer loaded with the KSPWFTTL synthetic peptide and conjugated with PE (Beckmann Coulter, Madrid, Spain).

All antibodies were purchased from BD-Biosciences (San Jose, CA, USA). Analyses were performed with FACS Calibur platform (BD Biosciences) and data were analyzed using FlowJo software (Tree Star Inc., San Carlos, CA, USA).

### RNA isolation and quantitative PCR analysis

Total RNA extraction from isolated CD8^+^ T cells was performed using the Maxwell® 16 Total RNA Purification Kit (Promega, Madison, Wisconsin, USA). The concentration and purity of samples were determined in a NanoDrop spectrophotometer with absorbance set at 260 and 280 nm (Thermo scientific, Wilminfton, USA). One microgram of RNA was retrotranscribed to cDNA with Moloney murine leukemia virus (M-MLV) reverse transcriptase from Promega, according to the manufacturer's instructions.

Real-time PCR was performed using Biorad reagents and to the manufacturer's specified protocol was followed.

Quantitative real-time PCR was performed by using specific primers for each gene. Transforming growth factor beta (TGF-β), Fw: *5′-cggcagctgtacattgac-3′* and Rv: *5′-tcagctgcacttgcaggagc-3′*. CD40 Ligand (CD40L), interferon gamma (IFNγ), Fw: *5′- tcaag tggcatagatgtggaa-3′* and Rv: *5′-tggctctgcaggattttcatg-3′*. Programmed cell death protein 1 (PD1), Fw: *5′-ac tggtcggaggatcttatg-3′* and Rv: *5′- atcttgttgaggtctccagg-3′*. Interleukin 10 (IL-10), Fw: *5′- ggacaacatactgctaaccg-3′* and Rv: *5′-aatcactcttcacctgctcc-3′*. Programmed death-ligand 1 (PD-L1), Fw: *5′-gatcatcccagaactgcctg-3′* and Rv: *5′-gcttacgtctcctcgaattg-3′*.Perforin 1 (*Prf1*) Fw: *5′-ag cacaagttcgtgccagg-3′* and Rv: *5′- gcgtctctcattagggagt tttt-3′*. Fas ligand *(Fasl)*. Granzyme A *(Gzma)* Fw: *5′-tg ctgcccactgtaacgtg-3′* and Rv: *5′- ggtaggtgaaggatagccac at-3′*. Granzyme B *(Gzmb)* Fw: *5′-ccactctcgaccctacatgg-3′* and Rv: *5′-ggcccccaaagtgacatttatt-3′*. Ribosomal Protein, Large, P0 (RPLP0), Fw: *5′-aacatctcccccttctcctt-3′* and Rv: *5′-gaaggccttgaccttttcag-3′*. As RPLP0 levels remained constant across different experimental conditions, this parameter was used to standardize gene expression. The amount of each transcript was expressed by the formula 2ΔCt (2ct(RPLP0) – ct(gene)), ct being the point at which the fluorescence rises significantly above the background fluorescence.

### Statistical analysis

Statistical analyses were performed using Prism 5 computer program (GraphPad Software Inc, San Diego, CA, USA). The survival data were represented in Kaplan-Meier graphs and the crossing curves were analyzed using the Renyi family of statistics. The SurvMisc package (https://cran.r-project.org/web/packages/survMisc/survMisc.pdf) was used to analyze the crossing curves with the weighted log-rank tests with the Fleming–Harrington class of weights. The remaining parameters were analyzed by two-way ANOVA, followed by the Bonferroni multiple comparison test. P<0.05 values were considered significant.
